# Pregnancy after Acute Coronary Syndrome: A Proposal for Patients' Management and a Literature Review

**DOI:** 10.1155/2013/957027

**Published:** 2013-06-06

**Authors:** Agnieszka Janion-Sadowska, Marcin Sadowski, Jacek Kurzawski, Łukasz Zandecki, Marianna Janion

**Affiliations:** ^1^Intensive Cardiac Care Unit, Świętokrzyskie Cardiology Center, 25-736 Kielce, Poland; ^2^Cardiac Catheterisation Laboratory, Świętokrzyskie Cardiology Center, 25-736 Kielce, Poland; ^3^2nd Departament of Cardiology, Świętokrzyskie Cardiology Center, 25-736 Kielce, Poland; ^4^Faculty of Health Science, The Jan Kochanowski University, 25-369 Kielce, Poland

## Abstract

Coronary artery disease complicates only 0.01% of all pregnancies. For this reason, more exhaustive data on the management of such cases is lacking. Even guidelines on management of cardiovascular disease in pregnant women are scarce focusing mainly on acute myocardial infarction. This is a complex issue involving thorough evaluation of cardiovascular status in each pregnant woman, assessment of risk for developing coronary complications, and close cooperation with obstetric teams. Safety data on typical cardiac drugs such as statins, angiotensin converting enzyme inhibitors, angiotensin receptor blockers, or novel antiplatelet drugs are also scarce and their effect on the developing human fetus is not well understood. We present a review on the management of such patients.

## 1. Introduction

Women of reproductive age are usually young and healthy and are at negligible risk of developing a serious health condition. Cardiovascular disease is estimated to occur in 0.4–4.1% of pregnant women, whereas coronary heart disease complicates only 0.01% of all pregnancies [1]. For this reason more exhaustive data on the management of such cases is lacking. In recent years a number of papers have been published on the treatment of myocardial infarction in pregnant women however, less attention has been paid to the management of pregnant women with a history of myocardial infarction or with stable angina pectoris. Even guidelines on management of cardiovascular disease in pregnant women are scarce focusing mainly on myocardial infarction [2]. However, it is not surprising because this is a complex issue involving thorough evaluation of cardiovascular status in each pregnant woman, assessment of risk for developing coronary complications, and close cooperation with obstetric teams. Safety data on typical cardiac drugs such as statins, angiotensin converting enzyme inhibitors, angiotensin receptor blockers, or novel antiplatelet drugs are also scarce and their effect on the developing human fetus is not well understood. Information on the course of pregnancy in women with coronary artery disease is collected mainly from single- or multiple-case studies [3–5] making it difficult to draw general conclusions. Moreover, not all clinical data are provided, but there are differences between cases in cardiovascular status, time elapsing after myocardial infarction, and mode of treatment (especially those reported in the 80s and in the 90s of the 20th century). One should also remember that the reported cases do not represent the entire population of pregnant women with coronary heart disease. There may be a number of cases, especially fatal cases, which have never been reported. Although coronary artery disease in pregnancy is rare, there is concern that the problem will occur more and more frequently. This is related to the social consequences of revolution in the recent years. Women stopped caring for their homes and started developing careers, frequently performing managerial jobs. The need to reconcile family and professional lives and the urge to prove themselves impose on women more than duty-related stress. In the 50s–70s of the 20th century, it was not acceptable for women to smoke. Now more and more women, especially the young ones, believing in women's equality in its broad sense develop this bad habit. Because of their wish to make and strengthen their careers many women choose to postpone pregnancy, frequently at the time when coronary risk factors are already present. Oral contraceptives especially in combination with smoking increase significantly the risk for developing myocardial infarction in young women, whereas assisted reproductive technology (*in vitro* fertilization, egg donation) helps to achieve pregnancy in older women with multiple coronary risk factors or already having coronary artery disease [6].

## 2. Pregnancy and the Cardiovascular System

Physiological hemodynamic and hemostatic changes occurring during pregnancy, delivery, and postpartum may significantly affect the cardiovascular system. Increased circulating blood volume, heart rate, and cardiac output increase myocardial oxygen demand. In patients with coronary lesions, this may induce anginal pain and even deterioration of cardiac function [7]. Birth-related pain and effort as well as sudden changes in blood volume and pressure postpartum may also significantly increase the risk for developing cardiovascular complications [1, 2, 6, 7]. Hypercoagulability in pregnancy as a result of increased fibrinogen and several blood clotting factors, increased platelet count, decreased protein S, activated protein C resistance, and weakened fibrinolysis due to impaired release of tissue plasminogen activator (tPA) and increased tPA inhibition may affect the course of coronary artery disease in pregnant women [2, 6]. The main causes of an abrupt coronary blood flow cessation during pregnancy are atherosclerosis (coronary plaque rupture or erosion followed), vasospasm, thrombosis, and coronary artery dissection. Atherosclerotic changes are responsible for 6–40% of acute MIs, mainly in older women with preexisting coronary risk factors like smoking, diabetes, arterial hypertension, dyslipidemia, and obesity [7–9]. Increased intrathoracic pressure and estrogen- and relaxin-related changes in connective tissue properties result in spontaneous coronary artery dissection in pregnant women more commonly than in general population (16–30% versus 0.28%). In most cases, it affects the left anterior descending artery during peripartum [7, 10, 11]. Smoking, alcohol abuse, family history of CAD, and migraine predispose to vasospasm induced by ergot derivatives, betamimetics, oxitocin, and bromocriptine as well as renin released from hypoperfused placenta and vascular hyperreactivity to vasoactive agents [11]. Other rare causes of AMI during pregnancy are emboli, vasculitis, myocarditis, connective tissue diseases, Ehlers-Danlos syndrome, pheochromocytoma, and sickle cell anemia [7–9]. Another pregnancy-specific risk factors are eclampsia and preeclampsia which have been shown to increase cardiovascular risk even up to one year after pregnancy [7, 11]. Ethnicity-related issues have also been investigated. In univariate models, Black women have the highest risk of pregnancy-induced myocardial infarction as compared to their Hispanic or White counterparts. In multivariate analyses, black race was not a significant factor which suggests that poor cardiovascular profile characterized by arterial hypertension, thrombophilia, diabetes, and smoking was responsible for increased prevalence of AMI [8].

## 3. Prepregnancy Counseling

In women of reproductive age with known coronary heart disease, a history of myocardial infarction or myocardial revascularization prepregnancy risk assessment is extremely important. There are many factors that influence the course of coronary heart disease in pregnant women. The changes that occur to a woman's body in pregnancy significantly affect the cardiovascular system; therefore, left ventricular systolic function, the condition of coronary arteries, time elapsed from myocardial infarction, and ischemic symptoms are very important. Prepregnancy assessment of the cardiac patient should include full medical history, physical examination, ECG at rest, exercise test, and an echocardiogram. If necessary, coronary CT angiography or even routine coronary angiography should be performed, women with significantly decreased ejection fraction, heart failure symptoms, angina pectoris, and advanced changes in coronary arteries should be discouraged from getting pregnant to avoid the situation of our patient with postmyocardial infarction heart failure in whom it was decided to terminate pregnancy because of worsening symptoms [1]. All medicines used by women planning to become pregnant should be reviewed to see which of the drugs should be continued, safely discontinued, or replaced by a safer alternative. A plan for cardiology and obstetric supervision and checkup visits during pregnancy should be made. Normal pregnancy care such as folic acid supplementation should not be forgotten either. Unfortunately many women do not consult their physicians when considering getting pregnant but present them with a *fait accompli*. Our patients with a history of acute coronary syndrome and myocardial revascularization did not seek medical (cardiac) advice before planning another pregnancy [3–5]. Of note, if a woman gets pregnant and is receiving potentially teratogenic drugs, she should undergo ultrasound imaging to check for possible malformations. Irrespective of the estimated risk, women with coronary heart disease should be under supervision of an experienced cardiology and obstetric team from very early pregnancy. In pregnant women coronary angiography (both invasive and CT scan) increases the risk of breast cancer [12]. Fetus exposed to irradiation has a greater risk of lethal malformation *in utero* and malignancy in the future [13]. This leads to conclusion that all examination with ionizing radiation must be avoided except for life-saving procedures, that is, primary percutaneous coronary intervention in AMI. However, proper shielding is mandatory and radial access is preferred to decrease fetal exposure [7, 12, 14].**  **Exercise testing, while not contraindicated due to several obstetric reasons, has a limited value in pregnant women due to a high false-positive results rate [14].

## 4. Management during Pregnancy 

Available evidence and our own observations show that coronary heart disease does not have a significant influence on maternal and child mortality, in the absence of heart failure, significant coronary abnormality, ongoing ischemia, or depressed ventricular ejection fraction [1, 15]. However, the condition may worsen and require urgent intervention. Furthermore, as mentioned before, fatal cases might have never been reported. For this reason, pregnant women should remain under supervision in order to immediately identify and eliminate potential risks.

## 5. Nonpharmacological Management 

Pregnant women, similar to all patients with coronary heart disease, should pay attention to lifestyle-related risk factors. Smokers when pregnant should be advised to stop smoking not only for mothers' sake but also because of negative effects of maternal smoking such as intrauterine growth retardation and premature delivery. Diet plays an important role, especially in women with hyperlipidemia as most lipid lowering drugs are contraindicated in pregnancy. It is recommended to reduce dietary intake of saturated fat in favor of unsaturated fats, including polyunsaturated fat [16]. Traditionally the best sources of unsaturated fats are oily fish such as salmon, tuna, herring, and mackerel. However, the American guidelines recommend reduced consumption of fish in pregnancy because they contain organic mercury [16]. Recent studies have demonstrated that increased marine pollution results in bioaccumulation of various toxins, for instance, methylmercury, in fish meat [17]. Its content depends on the species and age of the fish and fishing location. The highest methylmercury levels are found in large predatory fish such as tuna, swordfish, or shark. All fish for the market are inspected and the content of toxic substances does not exceed a critical value. However, low doses of mercury with no adverse effects in adults have been found harmful to the developing nervous system causing mental retardation [17]. Furthermore, very high levels of dioxins and dioxin-derived substances have been found in oily fish such as salmon or sprat. For this reason, dietary intake of saltwater fish should be reduced in pregnancy and breastfeeding. Calorie intake per day is also important in pregnancy. Although pregnant women have higher energy needs, they should be advised against overeating. Normal weight gain starts at 20 weeks and by the end of pregnancy should not exceed 20% of body mass prior to pregnancy. Sudden weight gain may be a sign of fluid retention caused by worsening heart failure or eclampsia. There are no recommendations regarding physical activity. Obviously, in case of significant left ventricular systolic dysfunction or coronary lesions physical effort, including daily life activities, should be limited. Women with preserved ejection fraction and normal coronary blood flow and with no other contraindications may perform moderate levels of individually tailored exercise. Because of unfavorable effects on fetal development, pregnant women should not be encouraged to drink alcohol, even in small “cardiac” amounts.

## 6. Pregnancy-Related Risk Factors

High volumes of plasma and increased demand for iron may induce anemia in pregnancy. The presence of increased myocardial oxygen demand may lead to the development of angina pectoris or worsening symptoms. It is therefore necessary to perform blood count tests and supplement iron. Gestational diabetes and pregnancy-induced hypertension may also affect negatively the cardiovascular system. Regular blood glucose and blood pressure measurements are important for early diagnosis and appropriate treatment. Women with diabetes and arterial hypertension prior to pregnancy require treatment modification, preferably when planning to become pregnant.

## 7. Pharmacotherapy

Traditional division into five categories (A, B, C, D, X) according to the Food and Drug Administration has been questioned for several years due to limitation in its ability to estimate the risk to benefit ratio. The new proposal of drug labeling requires a pregnancy and lactation subsection with detailed risk summary, clinical considerations, and a data section [18]. However, in many papers, it is continuously used for transparent recommendation [2, 7]. Briefly, they can be summarized as follows: category A—no risk to fetus in human studies, B—no risk to fetus in animal studies and there are no studies in pregnant women, C—adverse effect on fetus in animal studies and there are no human studies, D—evidence for human fetal risk exists, and X—fetal abnormalities and fetal risk in human or animal studies. Drugs labeled C or D may be considered in pregnancy due to their potential benefit despite known risk. The risk related to drugs labeled X substantially outweigh potential benefit and, therefore, should not be used [7, 18]. Unfortunately, depending on publication date there exist some discrepant reports on some drug classes, that is, heparins (Table 1).

## 8. Antiplatelet Drugs

Acetylsalicylic acid (ASA) is one of the oldest and best-described medicines used in secondary prevention of coronary heart disease. Low doses of acetylsalicylic acid are relatively safe in pregnancy, and teratogenicity has not been noted so far [2, 15]. However, one should remember that through inhibition of prostacyclin it may inhibit uterine contractions and prolong labor, increase blood loss, and cause premature closure of the ductus arteriosus. These effects, however, were noted with higher doses of acetylsalicylic acid than with those used for prevention of cardiovascular events [15]. ASA passes into breast milk but does not cause significant adverse effects [2]. It should, however, be emphasized that safety of ASA in early pregnancy has not been unequivocally established. In our pregnant patients after myocardial revascularization, with stable angina pectoris and preserved left ventricular contractility prior to pregnancy there were no negative effects of ASA. Using clopidogrel in pregnancy is controversial. Animal studies have not demonstrated teratogenicity, but there are no studies in humans and it is not known whether clopidogrel passes through the placenta or enters breast milk [2]. There are several reports on using clopidogrel in pregnancy, mainly as part of double antiplatelet therapy in women undergoing coronary stenting [15, 19, 20]. In some women double antiplatelet therapy was used only in the first trimester, in some only for a limited period of time, and in some throughout pregnancy until labor, and in 3 cases double antiplatelet therapy was withdrawn at one week before the planned delivery date and replaced with low molecular weight heparin. Fetal injury occurred in two cases. Santiago-Díaz et al. [20] described a woman who underwent PCI at 6-week pregnancy and who had been receiving antiplatelet therapy until delivery. Her baby had persistent foramen ovale, muscular ventricular septal defect, and mild mitral regurgitation. Shah et al. [19] reported intrauterine fetal demise in the 26th week of pregnancy in a woman undergoing CABG. Existing evidence does not permit one to draw reliable conclusions regarding safety of clopidogrel in pregnancy; however, it may be helpful in making decisions in individual cases. Due to the lack of data, the decision to give clopidogrel in pregnancy must always be based on cardiovascular risk assessment in mothers [15]. In the European guidelines, it is recommended to use clopidogrel only when it is absolutely necessary and for the shortest possible time [2]. The newest antiplatelet drugs such as prasugrel and ticagrelor are even less known. They have not been studied in pregnancy, and there is no information regarding their capability to pass through the placenta and into breast milk. In animal studies, prasugrel had no teratogenic effects, passed into breast milk, and did not affect fertility of adult individuals. However, due to a lack of data in humans, prasugrel in pregnant women may only be considered when the potential benefit to the mother exceeds any potential risk to the baby. Prasugrel is not recommended in breastfeeding women [2]. Ticagrelor at therapeutic doses did not have adverse effects on fetal development in animals. However, in doses 3–5 times higher ticagrelor had a negative effect on liver and skeletal development, caused intrauterine growth retardation, and after birth reduced offspring vitality and caused retardation of development. For this reason ticagrelor is not recommended in pregnant and breastfeeding women [2].

## 9. Beta-Adrenergic Blockers

Beta-adrenergic blockers have been shown to reduce mortality after myocardial infarction [16]. In patients with stable angina pectoris without myocardial infarction, they do not affect prognosis but they have antianginal effects. In the European guidelines, beta-blockers are recommended as relatively safe drugs for pregnant women. Most frequently used beta blockers (metoprolol, bisoprolol, carvedilol) pass through the placenta and into breast milk, are without proven teratogenicity, and may induce bradycardia and hypoglycemia in fetuses [2]. Atenolol is less safe when used in the first trimester it additionally causes hypospadias [2].

## 10. Statins

Statins have proven efficacy not only in lowering blood cholesterol levels but also in prevention of acute coronary syndromes [16]. However, their effect on human fetuses is not clear. Current guidelines recommend that statins should be withdrawn in pregnancy, and even as early as 3 months before planning to become pregnant [2, 21]. The European guidelines recommend that all hypolipemic agents should be withdrawn in pregnancy as their effect on human fetuses is unknown [2]. Bile-acid binding resins are regarded the safest in pregnancy because their effect is limited to the gastrointestinal lumen, they are not absorbed into the blood and cannot affect directly the fetus [2, 21]. Temporary interruption of the hypolipemic treatment during pregnancy in women with primary hypercholesterolemia does not have a significant negative effect on prognosis and should not affect the course of coronary heart disease [22]. However, taking into account the need for drug withdrawal before becoming pregnant, during breastfeeding and while pregnant with another child, the time without medication may significantly prolong. There are no studies exploring to what extent drug withdrawal may affect coronary risk profile in women. In pregnancy, total cholesterol levels rise significantly (by 30–50%), similar to LDL cholesterol and triglycerides (even 3-4-fold). This is associated with increased production of sex hormones and maternal and child demand. Pregnancy-related lipid disorders are not regarded as coronary risk factors [21–25]. However, in case of familial hypercholesterolemia, drug withdrawal may be harmful to the mother and the fetus. High cholesterol levels in pregnancy have a negative effect on fat infiltration into the aorta in newborn children as compared with mothers without familial hypercholesterolemia [21–25]. For this reason, it is recommended to replace statins with resins, and if ineffective or not tolerated, electrophoresis may be considered which has been found safe and effective in lowering total cholesterol and LDL cholesterol levels in pregnant women [21]. More and more investigations cast doubt on the teratogenic effects of statins on pregnancy outcome [22, 23]. Animal experiments provide equivocal results. Studies in rats and rabbits demonstrated adverse effect of fluvastatin and lovastatin on fetal development (skeletal deformities) [23, 24]. Simvastatin was not found to have teratogenic effects in animals, whereas adverse effects of atorvastatin emerged with doses that were toxic to mothers [23, 24]. The effect of statins on fetal development in humans is still not well understood. Some evidence shows that statins may interfere with the formation of the placental villi in the beginning of pregnancy and thus increase the risk of miscarriage. More lipophilic statins (lovastatin, simvastatin, atorvastatin) seem to pass easily through the placenta [23]. Data on statin safety in pregnant women can be found mainly in case reports and registries [21, 23, 25], however, not resolving the question of statin teratogenicity. The duration of treatment varies (mainly the first trimester), doses and type of drugs are different, and most abnormalities are not typical—they are most frequently developmental defects of the nervous, bone and skeletal systems, and ventricular and atrial septal defects. Adequate comparative groups are lacking. It is of importance because accompanying diseases such as diabetes mellitus or arterial hypertension increase the risk of congenital defects [23]. According to some investigators, the incidence of congenital defects in women using statins is similar to that in the general population, although children born to these mothers have lower birth weights. The lowest rate of congenital defects was found among women receiving pravastatin [23]. There is an increasing number of studies showing positive effects of statins, mainly pravastatin, on the course of eclampsia or antiphospholipid syndrome in animal models [24]. Other investigations in rabbit fetuses demonstrate negative effects of pravastatin on response to ischemia through impairment of nitric oxide metabolism [26]. At present statins are still contraindicated in pregnancy and breastfeeding, but perhaps the situation will change when the results of the ongoing studies are available.

## 11. Angiotensin Converting Enzyme Inhibitors

Angiotensin converting enzyme inhibitors (ACE-i) are used to treat arterial hypertension and heart failure. After myocardial infarction, ACE-i are recommended for secondary prevention in patients with decreased ejection fraction and/or accompanying diabetes. They pass through the placenta and into the milk. ACE-i impair the development of kidneys, lungs, and skull ossification they cause intrauterine growth retardation; and even intrauterine demise [2]. The teratogenic effects of ACE-i seem to appear if they are used in the second trimester [27, 28]. Data regarding the first trimester are unclear. Some studies show that ACE-i used in the first trimester do not increase significantly the risk of congenital defects in children, although it cannot be excluded that they increase the rate of miscarriage [27, 29]. Women of reproductive age should be warned of ACE-i teratogenicity. Women planning to become pregnant should discontinue treatment and ACE-i should be replaced with another antihypertensive agent. If a woman gets pregnant while receiving ACE-i, treatment should be discontinued and a fetal ultrasound should be performed [1].

## 12. Nitrates

Nitrates are commonly used in the management of patients with stable angina as well as acute coronary syndromes. They also may be used in pregnant women to relief angina and lower blood pressure in acute and stable cases. However, administration of glyceryl trinitrate or isosorbide dinitrate should be adjusted to hemodynamic status to avoid hypotension and placental hypoperfusion as well as fetal bradycardia [2, 7].

## 13. Heparins

Both unfractionated (UFH) and low molecular weight heparin (LMWH) do not cross the placenta. There is no evidence for fetal bleeding or teratogenicity. LMWH may be preferred over UFH due to more stable anticoagulation and lower risk of maternal bleeding complication and thrombocytopenia. UFH should be discontinued six hours and LMWH 24 hours before delivery [2, 7, 30].

## 14. Management of Worsening Symptoms

The symptoms of myocardial ischemia may be dangerous to the mother and child. Management should be tailored to the patient. Physical activity should be reduced, and pharmacotherapy should be optimized. If the symptoms persist or myocardial infarction develops, coronary angiography and myocardial revascularization should be considered [6].

## 15. Perinatal Management

There are no unequivocal guidelines for perinatal care. The controversy concerns mainly antiplatelet therapy whether it should be maintained, when it should be withdrawn, and when reinstituted. Patient safety should be the main determinant. There are reports on discontinuation of dual antiplatelet therapy a week before delivery and replacement with low molecular weight heparin [15]. No adverse coronary events were found in such women, but one should not forget that there is no evidence showing that stent thrombosis can be prevented using only LMWH. The mode of delivery depends on obstetric indications and the specific hemodynamic status. Stable patients can give birth naturally. Lateral position prevents from compression of the aorta and inferior vena cava, which in turn decreases the risk of hypotension and hemodynamic disorders during the pushing stage. It is recommended to use anesthesia and shorten the second stage of labor (forceps and vacuum) to “unload” the heart. Medications that cause vasoconstriction such as ergotamine and prostaglandins should not be used. Caesarean section is performed only for obstetric indications or if the patient is hemodynamically unstable [6].

## 16. Pregnancy as a Cardiovascular Test

In the American guidelines for the prevention of cardiovascular disease in women, pregnancy is regarded as a specific test predicting the development of coronary artery disease. It was demonstrated that women with preeclampsia in pregnancy were at higher risk for developing myocardial infarction, stroke, or thromboembolic event within the next 5–15 years as compared with women without this complication. Several investigators believe that preeclampsia is associated with preexisting endothelial dysfunction, and vascular or metabolic disorders which seem to be triggered during pregnancy that places a significant burden on the heart [31]. For this reason, the American guidelines recommend taking a detailed history of pregnancy complications in risk assessment [32]. However, there is no data regarding treatment of such women. Further studies are warranted to establish a reasonable diagnostic plan that would enable identification of women at highest risk and institute appropriate strategy to reduce the risk.

## 17. Conclusion

Data on the effects of pregnancy on the course of coronary heart disease is scarce. Available evidence indicates that in hemodynamically stable patients with preserved left ventricular systolic function pregnancy is possible and relatively safe. In contrast, women with poor hemodynamics resulting from significant left ventricular injury, reduced ejection fraction, and heart failure symptoms should be discouraged from becoming pregnant. Women of reproductive age with known coronary artery disease should be advised to consult the cardiologist before becoming pregnant. Pregnant women should be taken care of by an experienced obstetric and cardiac team. Acetylsalicylic acid in small “cardiac” doses and beta-blockers seem to be safe drugs in pregnancy. Angiotensin-converting enzyme inhibitors and statins should be discontinued during pregnancy and breastfeeding. Because of a growing interest and results of several studies, some statins may be approved for use in pregnancy. Pregnancy complications such as preeclampsia or gestational diabetes mellitus are regarded as cardiovascular risk factors.

## Figures and Tables

**Table 1:** Safety of medication used during pregnancy based on [2, 7].

Drug	FDA category
Acetylsalicylic acid	B
Clopidogrel	C
Beta-blockers	
Atenolol	D
Bisoprolol, labetalol, propranolol, metoprolol	C
ACE-inhibitors	D
Nitrates	B
Statins	X
LMWH	B
UFH	B, C

ACE: angiotensin-converting enzyme, LMWH: low molecular weight heparin, UFH: unfractionated heparin, and FDA: Food and Drug Administration. Categories described in the main.
